# Potential Impact of Seasonal Malaria Chemoprevention on the Acquisition of Antibodies against Glutamate-Rich Protein and Apical Membrane Antigen 1 in Children Living in Southern Senegal

**DOI:** 10.4269/ajtmh.14-0808

**Published:** 2015-10-07

**Authors:** Magatte Ndiaye, Khadime Sylla, Doudou Sow, Roger Tine, Babacar Faye, Jean Louis Ndiaye, Yemou Dieng, Aminata Collé Lo, Annie Abiola, Badara Cisse, Daouda Ndiaye, Michael Theisen, Oumar Gaye, Michael Alifrangis

**Affiliations:** Service de Parasitologie–Mycologie, Faculté de Médecine, Université Cheikh Anta Diop de Dakar, Dakar, Sénégal; Centre for Medical Parasitology, Department of International Health, Immunology and Microbiology, University of Copenhagen, Copenhagen, Denmark; Department of Infectious Disease, Copenhagen University Hospital, Copenhagen, Denmark

## Abstract

Seasonal malaria chemoprevention (SMC) is defined as the intermittent administration of full treatment courses of an antimalarial drug to children during the peak of malaria transmission season with the aim of preventing malaria-associated mortality and morbidity. SMC using sulfadoxine–pyrimethamine (SP) combined with amodiaquine (AQ) is a promising strategy to control malaria morbidity in areas of highly seasonal malaria transmission. However, a concern is whether SMC can delay the natural acquisition of immunity toward malaria parasites in areas with intense SMC delivery. To investigate this, total IgG antibody (Ab) responses to *Plasmodium falciparum* antigens glutamate-rich protein R0 (GLURP-R0) and apical membrane antigen 1 (AMA-1) were measured by enzyme-linked immunosorbent assay in Senegalese children under the age of 10 years in 2010 living in Saraya and Velingara districts (with SMC using SP+AQ [SMC+] since 2007) and Tambacounda district (without SMC (SMC−)). For both *P. falciparum* antigens, total IgG response were significantly higher in the SMC− compared with the SMC+ group (for GLURP-R0, *P* < 0.001 and for AMA-1, *P* = 0.001). There was as well a nonsignificant tendency for higher percentage of positive responders in the SMC− compared with the SMC+ group (for GLURP-R0: 22.2% versus 14.4%, respectively [*P* = 0.06]; for AMA-1: 45.6% versus 40.0%, respectively [*P* = 0.24]). Results suggest that long-term malaria chemoprevention by SMC/SP+AQ have limited impact on the development of acquired immunity, as tested using the *P. falciparum* antigens GLURP-R0 and AMA-1. However, other factors, not measured in this study, may interfere as well.

Although the incidence of malaria is declining in many parts of sub-Saharan Africa, it remains an important public health problem, especially in risk groups such as infants, children, and pregnant women. This heavy burden raises the need to optimize control tools and devise appropriate intervention schemes. Several studies have shown a sharp decline of the risk of malaria infections in these risk groups through the use of intermittent preventive treatment (IPT) with sulfadoxine–pyrimethamine (SP) in infants (IPTi),^1^,^2^ in pregnant women (IPTp)^3^,^4^ and by seasonal malaria chemoprevention with SP + amodiaquine (SMC/SP+AQ) of children between age of 1 and 5 years in regions with high seasonal malaria.^5^,^6^ In infants and young children, these prophylactic strategies have been shown to protect children from episodes of malaria, anemia, and death^5^,^7^,^8^ and have limited impact on drug resistance development.^9^,^10^ Since these strategies reduce parasite exposure this may compromise the acquisition of protective immunity. Correspondingly, studies have shown a decrease in antibodies to malaria antigens after chemoprophylaxis; however, this may simply represent less parasite exposure rather than an actual loss of protective immunity.^11^,^12^

In Mozambique, chemoprophylaxis with SP did not significantly modify the development of natural immunity in infancy.^13^ In Ghana, antibodies against various *Plasmodium falciparum* antigens were significantly lower in children treated once with SP than in controls.^14^ Thus, despite its beneficial impact, mass implementation of malaria chemoprophylaxis raises concerns on whether naturally acquired immunity in treated individuals develops as in untreated ones (whether there is a rebound effect). The long-term effect of SMC/SP+AQ on immunity development in areas where this strategy has been routinely used for several years is not well documented. Thus, the aim of this study was to determine the potential impact of SMC/SP+AQ after the strategy has been implemented for 3 years on malaria immunity development in Senegalese children.

Samples were collected during a cross-sectional survey in 2010 involving children under 10 years of age living in three health districts located in southern Senegal where malaria transmission is highly seasonal (see Figure 1

**Figure 1.**
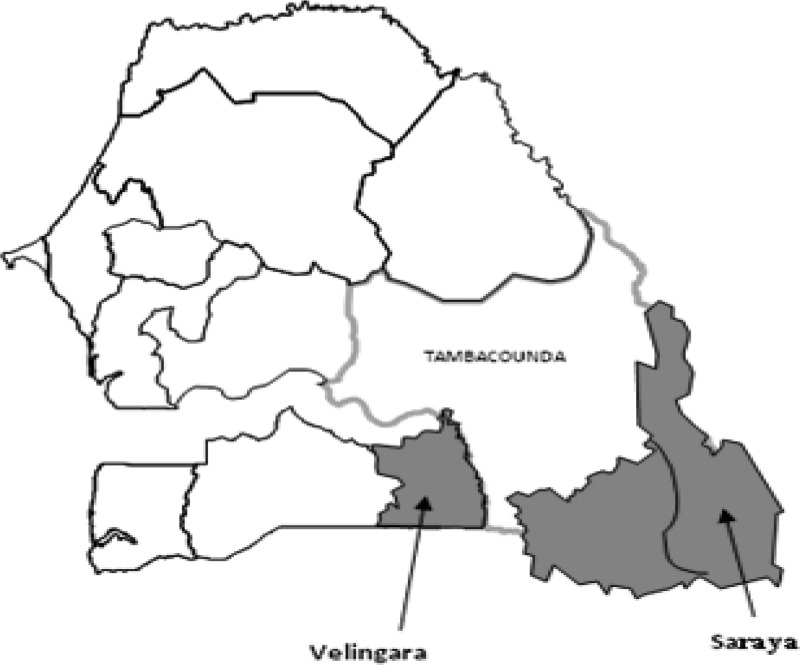
Map of Senegal showing the study sites. Saraya and Velingara are districts were seasonal malaria chemoprevention (SMC) has been implemented since 2007, whereas in Tambacounda, SMC has not been implemented and thus, function as a control district.

). Two of these districts (Saraya and Velingara) have implemented SMC with one dose of SP+AQ on day 1 (given by the community health workers), followed by two doses of AQ on days 2 and 3 for 3 months (August–October) (see Table 1) since 2007, whereas SMC was not implemented in Tambacounda district during this period and thus, function as a control district. Both areas have received universal coverage of bed nets. The latest data on malaria transmission occurring from June to November in this area have shown that the mean entomological inoculation rate (EIR) was 264 infected bites per year in 2003.^15^ Before blood sample collection, written informed consent was obtained from parents or guardian of each child. The study was approved by the Ethics Committee of Senegal named Comité National d'Ethique pour la Recherche en Santé (CNERS). During the study, if children presented to health posts with symptoms consistent with mild symptomatic malaria (temperature > 37.5°C) and a positive *P. falciparum* histidine-rich protein II rapid diagnostic test (Standard Diagnostics, Inc.; www.standardia.com), they were offered standard arthemisin combinaison therapy (ACT) first-line treatment (artesunate–amodiaquine) while children with severe malaria were referred to the nearest health district hospital. Finger-prick blood samples were collected from each study participants and blotted onto pre-labeled chromatographic filter paper (Whatman 3M; Maidston, Life Sciences United Kingdom), and stored with silica gel at 4°C until the serological analyses. Thick and thin blood films were also done for microscopic identification of *Plasmodium* species.

Blood thick smears were stained with 5% Giemsa, and parasite density was determined by counting the number of asexual parasites per 200 white blood cells, and calculated per microliter using the following formula: number of parasites × 8,000/200, assuming a white blood cell count of 8,000 cells/μL. Absence of malaria parasites in 200 high-power ocular fields of the thick films by two microscopists were considered as negative.

A total of 1,578 children under 10 years were enrolled in districts with SMC (Saraya and Velingara) and similar number of children under 10 years were enrolled in the district without SMC (Tambacounda). Malaria prevalence by microscopy was 7.3% (116/1,578) and 10.1% (159/1,578) in SMC+ and SMC− districts, respectively (*P* = 0.012).

From the 1,578 collected filter paper samples in each district, a randomization list was generated and a subsample of 372 (186 from the SMC+ group, mean age 6.2 ± 1.4 [93 positives + 93 negatives by microscopy] and 186 from SMC− group, mean age 6.3 ± 1.5 [93 positives + 93 negatives by microscopy]) was selected for serological analysis. The 372 filter paper samples were extracted as described (16). Antibody titers were measured by indirect enzyme-linked immunosorbent assay as described in reference,^17^ using the *P. falciparum* glutamate-rich protein R0 (GLURP-R0) and *P. falciparum* apical membrane antigen 1 (AMA-1) recombinant proteins.^18^ Positivity of samples was defined as optical density values above the mean of negative controls plus three standard deviations.

For both antigens, total prevalence of IgG seropositive responders beyond the calculated thresholds were higher in the SMC− compared with the SMC+ group (for GLURP-R0: 22.2% versus 14.4%, respectively, *P* = 0.06; for AMA-1: 45.6% versus 40.0%, respectively, *P* = 0.24). Similarly, regarding the crude IgG response (measured as arbitrary units), they were significantly higher in the SMC− compared with the SMC+ group (for GLURP-R0: 0.085 ± 0.083 versus 0.065 ± 0.064, *P* < 0.001 and for AMA-1: 0.225 ± 0.216 versus 0.124 ± 0.177, *P* < 0.001). When subdividing the groups into those that were positive or negative for *P. falciparum*, respectively, among the *P. falciparum* negative samples, the mean level of antibody response against GLURP-R0 was significantly higher in the SMC− as compared with SMC+ group (0.089 versus 0.025, *P* < 0.001); however, this was not significant regarding AMA-1 (0.021 versus 0.007, *P* = 0.75). Similarly, for the *P. falciparum* positives samples, the IgG response was significantly higher in SMC− group (GLURP-R0 = 0.308 ± 0.29; AMA-1 = 0.13 ± 0.09) compared with SMC+ group (GLURP-R0 = 0.182 ± 0.22; AMA-1 = 0.095 ± 0.07), *P* < 0.001 and *P* = 0.001 for GLURP-R0 and AMA-1, respectively (see Figure 2

**Figure 2.**
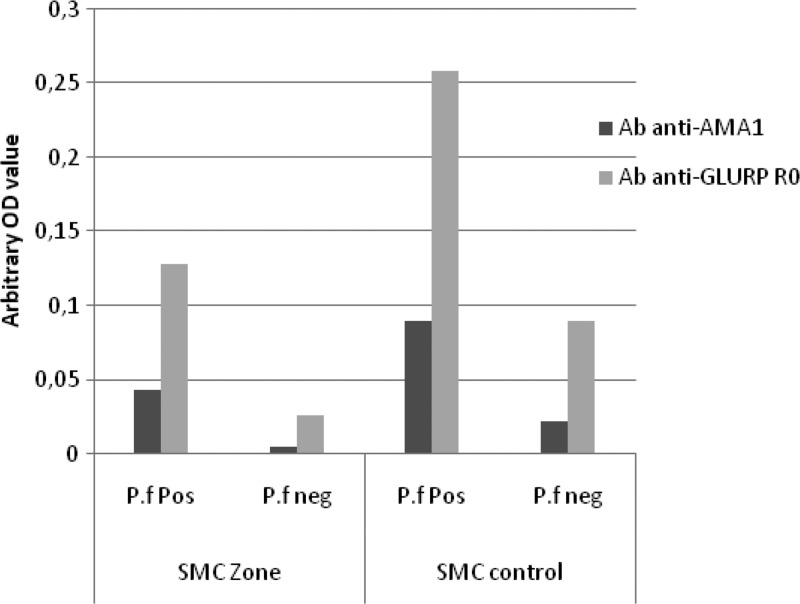
Antibodies (Ab) response in children under 10 years of age in area with and without seasonal malaria chemoprevention (SMC). P.f pos: *Plasmodium falciparum* positive by microscopy; P.f neg: *P. falciparum* negative by microscopy; SMC zone: area with SMC intervention; SMC control: area without SMC.

).

By comparing antibody responses to *P. falciparum* antigens in children under 10 years of age living in areas where SMC/SP+AQ were implemented for 3 years with an area without SMC, this study showed that for both *P. falciparum* antigens GLURP-R0 and AMA-1, total IgG response were higher in the district without SMC implementation. Similar results were found in Senegal by Boulanger and others^19^ where they showed that children receiving SMC had a slightly lower level of anti-*Plasmodium* schizont antibodies compared with non-treated control children after 8 months of implementation. Thus, the lower falciparum-specific antibody level noticed in the districts with SMC/SP+AQ most likely represent a lower development of acquired immunity toward malaria, and may be directly due to the SMC strategy. However, this difference could also be affected by differences in malaria transmission between these districts. Malaria transmission data (EIR) were not available during our study period, which is a limitation of our study. Our observations show that long-term malaria chemoprevention by SMC/SP+AQ may have limited impact on the development of antibody response against *P. falciparum* antigens such as GLURP-R0 and AMA-1; however, other factors may interfere such as heterogeneity of exposure and higher previous exposure that are significant predictors of higher antibody responses.

## Figures and Tables

**Table 1:** SP+AQ dosages in SMC

	Day 1 (TDO)	Day 2	Day 3
3–11 months	½ cp SP + ½ cp AQ	½ cp AQ	½ cp AQ
12–59 months	1 cp SP + 1 cp AQ	1 cp AQ	1 cp AQ
5–9 years	1½ cp SP + 1½ cp AQ	1½ cp AQ	1½ cp AQ

AQ = amodiaquine; SMC = seasonal malaria chemoprevention; SP = sulfadoxine–pyrimethamine; TDO = treatment direct observed.
